# Ethical considerations related to virtual visiting for families and critically ill patients in intensive care: a qualitative descriptive study

**DOI:** 10.1186/s12910-024-01130-z

**Published:** 2024-11-09

**Authors:** Kirsty Clarke, Karen Borges, Sultan Hatab, Lauren Richardson, Jessica Taylor, Robyn Evans, Bethany Chung, Harriet Cleverdon, Andreas Xyrichis, Amelia Cook, Joel Meyer, Louise Rose

**Affiliations:** 1 Imperial College London, London, UK; 2 Florence Nightingale Faculty of Nursing, Midwifery and Palliative Care, King’s College London, London, UK; 3 Palliative Medicine Cicely Saunders Institute, King’s College London, London, UK; 4 Guy’s and St Thomas’ NHS Foundation Trust, London, UK

**Keywords:** Telecommunications, Telehealth, E-health, Critical care unit, Visiting family, Visiting

## Abstract

**Background:**

During the COVID-19 pandemic, virtual visiting technologies were rapidly integrated into the care offered by intensive care units (ICUs) in the UK and across the globe. Today, these technologies offer a necessary adjunct to in-person visits for those with ICU access limited by geography, work/caregiving commitments, or frailty. However, few empirical studies explore the ethical issues associated with virtual visiting. This study aimed to explore the anticipated or unanticipated ethical issues raised by using virtual visiting in the ICU, such that healthcare professionals can be informed about how to carry out virtual visits ethically, safely and productively.

**Methods:**

We used a descriptive exploratory qualitative research approach recruiting a convenience sample of newly-graduated junior doctors facilitating ICU virtual visits in a tertiary academic centre. Eight newly graduated junior doctors, seven female and one male, aged 23–27, participated in semi-structured interviews. We analysed transcripts using an inductive coding approach.

**Results:**

Five overarching themes emerged. Two of the themes namely, ‘fulfilling a moral instinct to connect families’ and ‘promoting autonomy’, arose from participants’ descriptions of how virtual visits aligned with healthcare standards and practices they considered ethical. Three further themes, ‘preserving dignity and privacy’, ‘managing emotional distress’, and ‘providing equitable access’ to virtual visiting technologies, highlight how virtual visits might exacerbate ethical issues related to family communications.

**Conclusion:**

Virtual visiting may potentially both ameliorate and exacerbate aspects of ethical healthcare delivery for ICU patients and family members. ICU team members should consider unique ethical considerations related to using virtual visiting. We recommend virtual communications skills training for staff and advocate for the use of easily accessible educational resources for families who wish to visit critically unwell patients remotely.

**Supplementary Information:**

The online version contains supplementary material available at 10.1186/s12910-024-01130-z.

## Introduction

ICUs are ethically charged environments where life and death decisions are made daily in emotional situations that often involve legally incompetent patients [1]. The presence of families at the bedside of critically unwell patients is valued by staff and considered central to high-quality patient care and informed decision-making [2]. Professional society-endorsed guidelines for family-centred care recognise the role of family members in improving patient outcomes, staff experiences, and patient rehabilitation [3]. Studies that seek the perspectives of family members and patients on the role of families in the ICU indicated family involvement in care decisions, transparent clinical updates, and the opportunity for connection during times of emotional distress as key priorities [4]. Yet, the COVID-19 pandemic led to wide-scale in-patient visiting restrictions, exponentially increasing the demand for virtual visiting technologies that allowed patients to spend time with their families safely via video link [2]. Virtual visiting was particularly sought for patients who were too incapacitated to video-call their family using their own devices. Furthermore, these patients could not speak to relatives over the phone due to the presence of an endotracheal tube for mechanical ventilation [5]. In cases where patients were heavily sedated to allow such life-saving interventions, the video link meant families could spend time with such patients as if they were sitting at their bedside [6, 7].

In the UK, integration of virtual visiting into the care of ICU patients initially took place through a trial-and-error process before clear guidelines or an ethical framework could be put in place [8]. Anticipated ethical issues relating to patient consent were first managed by applying the existing guidance for in-person visits to virtual visiting. In March 2020, the UK Intensive Care Society published a rapid update to their guidance for managing COVID-19 critical illness, which recognised the use of videoconferencing technology [9]. However, it did not provide clarity on whether virtual visits should be carried out when considered by the ICU team in the best interests of unconscious patients [8]. Concerns regarding video link security, access to private video-calling accounts and safe patient data storage were raised [6]. One secure solution used widely across the UK was a bespoke ICU virtual visiting solution using the e-platform aTouchAway™ (Aetonix, Canada), already approved for use in healthcare [2, 5, 10, 11]. Similarly, to ensure virtual visits did not place additional demands on overstretched ICU staff and resources, many centres relied on charitable donations of video-call-enabled tablet devices and created dedicated virtual visiting teams [2, 5, 12].

In the post-pandemic era, there is an ongoing role for virtual visiting when in-person visiting is not feasible due to geographical constraints, work/caregiving commitments, frailty, ill health, or incapacity [6, 11]. However, for virtual visiting to be sustainably integrated into the care of critically unwell patients, ethical issues associated with this practice must be discussed and empirically investigated. To our knowledge, there has been no empirical investigation into the ethics of virtual visiting technology in the ICU setting. This study aimed to explore the anticipated or unanticipated ethical issues raised by using virtual visiting in the ICU, such that healthcare professionals can be informed about how to carry out virtual visits ethically, safely and productively.

## Methods

This study used a descriptive exploratory qualitative research approach [13]. We used convenience sampling methods, inviting participation from all newly graduated junior doctors working as part of a virtual visiting team at a central London (UK) teaching hospital. The recruitment period (April and July 2020) covered the height of the first wave of the COVID-19 pandemic in the UK when widespread use of virtual visiting technology was introduced. One research team member (AC, a female doctor working in palliative care with post-graduate qualitative research methods training) conducted in-person semi-structured one-on-one interviews using an interview guide iteratively developed by the research team (see Supplementary Material). After conducting eight 35–40 min interviews with eight newly graduated junior doctors (seven female and one male, with a mean age of 26), sufficient informational power was achieved, as the data collected provided rich and relevant insights to address the research question [14, 15].

Interviews were digitally recorded and professionally transcribed. The resulting transcripts were analysed using an inductive coding approach [16]. Each transcript was read and reread, with initial labels of one to two coding words placed in the transcript margin to identify ideas and/or concepts of interest. These codes were reviewed, and groups of similar ideas and concepts were collated. We undertook check-coding, as described by Miles and Huberman [16], during the initial transcript coding. Inter-coder agreement was achieved between the authors following comparison and discussion/debate over the categories. These groupings were then named as initial themes using one to two words that captured the essence of the content. These groups formed the preliminary themes. The content of each preliminary theme was reviewed to ensure the ‘best fit’ of content [16]. Groups containing similar content were combined to create the final themes. No participants withdrew, and therefore, data from all participants were included in the analysis.

To enhance trustworthiness and credibility, themes were tested by a process of member checking, with participants reviewing the data interpretations and confirming that they reflected the data they provided [17]. Additionally, the researchers engaged in peer debriefing, where data interpretation was refined iteratively through discussion [18]. Multiple rounds of coding also ensured the consistency and rigour of the data analysis, enhancing the dependability of results [16].

Importantly, all authors without extensive qualitative research experience were guided by those who had previous qualitative research training (KC, LR, AC, AX) to focus on reflexivity, enabling all researchers to recognise their pre-understanding and mitigate their biases [19]. All authors had a clinical background with exposure to a UK-based intensive care setting. The study interviewer, AC, had previous ICU and palliative care experience, which aided in rapport building and dialogue quality [14]. Moreover, conducting interviews while the participants were engaged in virtual visiting activities generated in-depth data by allowing participants to recall detailed descriptions embedded in the ICU context [14].

## Results

Analysis of the interviews revealed five major themes. Two of the themes arose from participants’ descriptions of how virtual visits helped ameliorate ethical dilemmas that arise in the ICU: ‘fulfilling a moral instinct to connect families’ and ‘promoting autonomy’. Three themes identify unanticipated ethical issues which may be exacerbated by the use of virtual visits depending on how they are conducted and resource availability, such as ‘preserving dignity and privacy’, ‘managing emotional distress’, and ‘providing equitable access’ to virtual visiting technologies.

### Fulfilling a moral instinct to connect families

Participants described a sense of moral satisfaction arising from providing patients and families with an opportunity to connect at a time when this would not have otherwise been possible. They explained how the role aligned with the overarching duty of their profession to make people “feel better” and caused them to feel privileged to be in their role as healthcare workers.

*“That’s one of the reasons I went into medicine was to try and make people feel better when things are bad. It was just an incredible opportunity”* (Participant 7).

*“I think that that is such privilege and the amazing thing about this whole project*, *I guess. So*, *yeah…The human connection you get is amazing and so rewarding”* (Participant 4).

Participants also described instinctual actions they took to enhance the connection between families and patients virtually, such as surrogate hand holding (where the participant would hold the hand of the patient on behalf of a family member).

*“she just kept saying*, *“I’m holding your hand. I’m holding your hand.” And so then I held the patient’s hand and said*, *“It’s okay*, *I’m holding her hand for you” and then the parents were just crying and crying and they were like*, *“We’re so grateful. We’re so grateful”* (Participant 1).

### Promoting autonomy

Often, participants described how the virtual visits allowed adjustments to be made so that patients.

who were too unwell to speak over the telephone could still express their thoughts and personalities non-verbally. This supported patients’ autonomy by facilitating self-expression.

*“the delight on their face as they could communicate with him–for the patient to be interacting and*.

*sort of joking around as well*, *like saying some things like “ohh*, *it’s like a hotel in here”*, *Like writing*.

*it on a board. It’s just quite sweet. That’s when it’s really worthwhile”* (Participant 4).

For patients receiving end-of-life care, participants described how virtual visits facilitated families’ autonomy in deciding how to spend their final moments with the patient.

“the patient wasn’t able to speak because of the trache, but he was mouthing responses. So I.

remember the last thing he said was she told him that she loved him, and he just mouthed with.

whatever strength he could that he loved her…I think not long after, he passed away.” (Participant 1).

**“**It didn’t matter how different the people were or how they used the call or, you know, whether or not they sang or got a priest or talked to their relative or whatever…” (Participant 2).

### Preserving dignity and privacy

Participants described their thoughts and concerns regarding how the privacy of patients and their family members could be preserved while conducting virtual visits. For example, when patients were too weak to hold the video call device, it was held for them. The virtual visit would be set up so that only the patient was in the family member’s field of view and the participant holding the video call device could not be seen, however they were still witness to the conversation.

*“in our role*, *we’d hold the tablet*, *and we’d swap the camera so that from the relative’s point of view*, *they can only see the patient. So for them they kind of forget that you’re there.”*(Participant 7).

Participants reported feeling unsure whether the relatives realised their conversations were audible to staff, especially as some conversations were personal to families and patients. The physical dependence of heavily sedated or unconscious patients during video calls, along with the helplessness of families relying on staff to facilitate these interactions, underscored the importance of protecting both from vulnerability. Participants highlighted the need to be mindful of preserving the dignity and levels of exposure for patients and families during video calls.

*“Obviously being on loudspeaker*, *everyone can hear what is being said and everyone can hear the*.

*content of it.”* (participant 3).

*“in the community*, *you never see them cry. Like these really strong men would then fall apart on the phone and*, *again*, *like I said at the beginning*, *about them not realising that they could see– that you could see them.”*(Participant 2).

Consent for virtual visits was either taken before a patient lost capacity due to their illness or consent.

was granted by a family member or member of the ICU team in the patient’s best interests. Some participants reflected on the act of facilitating a virtual visit between a heavily sedated patient and their family, being in line with what they would have wanted as a patient.

*“They were unconscious*, *and sometimes you think*, *would I like my loved ones to see me like that?*

*With all the tubes? But then you think*, *I can’t imagine them not being there with you”* (Participant 7).

### Managing emotional distress

On occasions when a family member became distressed, participants expressed regret that they could not reassure them the same way they would with an in-person visit. Video calling placed greater emphasis on verbal reassurance. Finding the correct words was challenging for participants when families were distressed.

*“you want to comfort them*, *but what can you say over a video call? “I can see you’re really upset. I hope you’re okay” But clearly they’re not okay because they’re crying…”* (Participant 3).

Participants described how, at times, the helplessness they felt when unable to reassure families during the calls, impacted upon their own well-being. As family members joined calls from their home environment, participants gained insight into the patient’s and family member’s social context and private life. Though this aided rapport building, it also made it more difficult for participants to manage their emotions. Despite experience working in the ICU setting, participants described how video calls could cause them to experience emotional breakdowns in the workplace for the first time.

*“she was crying saying “please sir*, *please*, *please help me. Help my daughter” And she said “I cannot lose another one” And*, *uh*, *I found out that her husband passed away in January and*, *uhm*, *it was*, *I– after that call and not being able to help. Feeling so helpless in hearing somebody that wouldn’t be older than my grandmother to beg down the phone like that. And*, *uh*, *I think it was the first time in my life that I’ve ever walked into the corner of a ward and just cried.”* (Participant 1).

When a video call had to be ended abruptly to provide emergency care, participants often struggled with how to communicate this to the family. They described making quick decisions in the moment that they later regretted. Compared to an in-person visit, during which a family member might pick up on cues from the staff and ICU environment that their relative is deteriorating, during virtual visits, this responsibility fell entirely on the participants. As a result, virtual visits placed a greater emphasis on competency in verbal communication skills, with clear and sensitive explanations crucial for conveying the need to interrupt a video call due to the severity of the patient’s condition.

*“I was just about to press the button to turn the camera around so they can see him and he went into*, *like*, *real bradycardia all of a sudden.And then the nurse kind of made the signal like*, *“Cut the call*, *cut the call”. So I had to just make up this excuse on the spot about how the ward round had come and hang up the call. I felt awful afterwards…”*(Participant 4).

### Equitable access to virtual visiting technology

During the pandemic, the number of patients who required virtual visits surpassed the resources and time available to the virtual visiting team. The critically unwell state of patients, combined with the dependence of families on virtual visiting technology, highlighted the need for clinical and ethical guidance on how virtual visiting should be integrated into clinical services. Virtual visiting guidelines were perceived as vital to prevent staff from feeling isolated in their decision-making.

*“…There were times where we were doing like 50 odd calls a day. And that was between a team about 5 or 6 of us.”*(Participant 4).

*“I’ll always remember– There was this patient who got admitted and it was right at the end of the day and I’d done the whole ward and it was already past the time that I was supposed to go home*, *and I just thought I’ll have to do that first thing in the morning and then I came in again the next day and the patient had died. And I just felt terrible that I’d never done it.”* (Participant 2).

One participant described language barriers and the family member’s confidence in using technology affecting how well they could connect with a patient. They identified strategies used to overcome such barriers.

*“I managed to speak to this guy and explain exactly how to set up the app and then finally got like his– he spoke to the wife*, *and then finally*, *I’ve got the wife on the call and it was just this amazing moment because it’s taken me a whole week to finally get this lady. And I’m there and I was like*, *“Maria*, *you’re here*,*” and she was like*, *“I’m so happy I can see you*,*” or words to that effect in broken English”*. (Participant 3)

## Discussion

This study aimed to understand the ethical implications relating to virtual visits such that healthcare professionals can be empowered to carry out virtual visits safely, ethically, and effectively. Our results have revealed two themes related to how virtual visits may ameliorate ethical dilemmas in the ICU, namely ‘‘fulfilling a moral instinct to connect families’ and ‘promoting autonomy’. Three further themes highlight unanticipated ethical issues which may be exacerbated by the use of virtual visiting depending on how it is managed and resource availability such as ‘preserving dignity and privacy’, ‘managing emotional distress’, and ‘providing equitable access’ to virtual visiting technologies.

The findings of this study corroborate existing evidence that virtual visiting fulfilled what many healthcare professionals experience as a moral instinct to connect critically unwell patients in the ICU with their families [20, 21]. For participants in this study, fostering therapeutic family connections virtually when in-person visiting was unavailable was seen to fit within the broader ethical responsibility for healthcare professionals to act benevolently and “do good” [22]. Importantly, the moral instinct that healthcare professionals feel to connect families is particularly salient in the context of end-of-life care when, even in the absence of visiting restrictions, there is not always enough time for some family members to arrive at a patient’s bedside [21]. Telephone communications are insufficient in these situations, as many critically unwell patients cannot communicate verbally. Thus, video-call-enabled devices are instrumental in giving patients and families the freedom to act autonomously and take ownership of how they spend their final moments. Building upon evidence published elsewhere, our findings indicate that it is important for healthcare professionals to recognise virtual visits as a viable method for providing patients dying in the hospital with a “good death”, especially when home or hospice transfer cannot be achieved, which is common in the critically ill [5].

Interestingly, our participants did not describe concerns related to data security or network/technical faults when using video-call devices, although this may be because participants knew they were using a virtual visiting solution bespoke to the ICU that was secure and SIM-enabled to avoid difficulties associated with hospital wifi. Instead, unanticipated ethical dilemmas arose regarding the privacy of patients and families when virtual visits were taking place. Primarily, participants expressed uncertainty over whether relatives were aware that staff outside of the video’s field of view could still overhear the conversations they were having with patients. Notably, studies exploring virtual visiting from the perspective of families have not identified similar concerns over privacy [2]. Furthermore, there may be a disconnect between families and ICU staff regarding the expectations, concerns and priorities of virtual visiting [6]. For example, while ICU staff have reported reluctance to engage in virtual visits, families do not share these concerns and instead prefer frequent interactions with staff during virtual visits [7]. Undoubtedly, our findings advocate for an approach where virtual visits are set up, ensuring staff are visible at the patient’s bedside (as expected during an in-person visit) [7]. Nonetheless, the patient and family perspective on relationships between patient exposure and preserving dignity for vulnerable ICU patients must be central to virtual visiting practices [23]. The existing evidence in this area emphasises the role of an “ethos” of compassionate care, with maintaining dignity going beyond technical tasks to include a moral and emotional connection with patients [24–26]. Due to the physical dependence and vulnerability of critically ill patients, a focus on interpersonal connection and empathy can alleviate a patient’s sense of exposure and loss of control [27]. Concerning virtual visiting, time should be taken to adjust bedding and ensure appropriate body coverage, pain relief should be provided, and any personal hygiene concerns should be addressed a video call is made. These simple patient-centred methods for maintaining dignity should also be emphasised when developing virtual visiting guidelines [28].

With the view to prevent the emotional distress of families during virtual visits, our findings underscore the role of clear guidance for families and healthcare professionals as a prerequisite for virtual visits. For example, explaining to families that visits may be cancelled at short notice is important to allow healthcare professionals to provide routine care (e.g., bathing, suctioning of endotracheal tube) and emergent procedures (e.g., intubation and central line placement) [8]. However, while the participants in this study described feeling worried about the distress expressed by families upon seeing patients in a critically unwell condition, virtual visits have been found to have a net positive effect on family members’ well-being overall [10]. Furthermore, our findings indicate that despite adequate informed consent, negative emotions can be expressed by family members as part of a natural grief reaction, emphasising the need for healthcare professionals conducting visits to possess skills in grief management and post-visit debriefing [21].

Alongside ensuring virtual visits do not cause emotional harm to families, it is important to ensure virtual visits do not negatively impact healthcare professional well-being [21, 29]. The emotional burden expressed by our participants while conducting virtual visits remains poorly understood [6] but may relate at least in part to the physical barrier created by virtual technologies [20, 29]. Our findings suggest that physical separation can make staff feel helpless in their efforts to comfort families and less confident in reading social cues. However, family members do not report similar concerns over bedside manner during virtual visits [5, 6]. Going forward, virtual communication skills training should be widely adopted in post-graduate and undergraduate settings to enable healthcare professionals using virtual visiting to feel more comfortable handling sensitive situations virtually [11, 21]. Such training is likely to become increasingly important as video-conferencing technologies are relied upon more in healthcare [30].

Regarding equitable access to virtual visits, our participants described feelings of guilt relating to the inability to provide virtual visits to all patients when requested, given to the large volume of patients during the pandemic and the limited time and resources to meet this need. While the demand for virtual visiting services has reduced, some clinicians have expressed concerns that virtual visits are too time and resource-intensive to be equitably and sustainably integrated into usual ICU visiting practices [8]. Encouragingly, numerous studies have demonstrated that virtual visiting using user-friendly software can be carried out in the ICU in a manner that does not place time pressures on ICU staff [7]. Moreover, as the healthcare industry increasingly recognises the wide applications of virtual technologies [30], we look forward to a time when video-call-enabled devices, which are affordable and user-friendly, will be more freely accessible.

Strengths of this study include a detailed exploration of the ethical issues relating to virtual visiting for critically unwell patients, which, to our knowledge, has not been empirically investigated. Similarly, we ensured trustworthiness and credibility of our data interpretation by employing member checking involving all participants. Applying the principles of transferability, our findings are useful in other ICU settings/contexts. Limitations of this study include the use of a convenience sample, which may have biased results due to the under-representation of some participant groups. However, virtual visits conducted in other centres using different infrastructure or methods may have raised different ethical issues than we identified, as our participants were employed at a single large tertiary centre based in London. We achieved informational power with a relatively small number of interviews (eight). The small sample is reflective of the high quality of dialogue achieved as conducting the interviews in the hospital between shifts allowed participants to vividly recall and provide detailed descriptions of their experiences [14, 15]. Additionally, the timeliness of these interviews, conducted during the COVID-19 pandemic, contributed to the depth and richness of the data, capturing the extraordinary and globally challenging conditions faced by intensive care workers during this period.

We recommend that further research on this topic be conducted in other contexts, such as ICUs conducting “business as usual” outside of pandemic work and alternative settings, such as paediatric ICUs. A future study combining the viewpoints of family members and ICU team members would be valuable in gaining a collaborative understanding of the ethical issues raised by virtual visiting and help build an ethical framework to support its use. Our recommendations for practice include easily accessible virtual communication skills training for ICU healthcare professionals and clear and easily accessible educational resources for families who wish to undertake virtual visiting.

## Conclusion

Virtual visiting may potentially both ameliorate and exacerbate aspects of ethical healthcare delivery for ICU patients and family members. Virtual visiting raises unique ethical considerations which should be addressed, as this technology is now used as an adjunct to in-person visiting outside of pandemic conditions. Key issues highlighted by this research include the measures which should be taken to preserve families’ privacy and limit emotional distress. Hence, we recommend virtual communications skills training for staff and advocate for the use of easily accessible educational resources for families who wish to visit critically unwell patients remotely.

## Electronic supplementary material

Below is the link to the electronic supplementary material.

Supplementary Material 1

## Data Availability

The qualitative datasets generated and/or analysed during the current study are not publicly available but are available from the corresponding author on reasonable request.
