# Supplementary material for: Ethical considerations related to virtual visiting for families and critically ill patients in intensive care: a qualitative descriptive study
Source: BMC Med Ethics. 2024 Nov 9;25:126. doi: 10.1186/s12910-024-01130-z (PMC11549751; doi:10.1186/s12910-024-01130-z)
Supplement: Supplementary file 1 — Supplementary Material 1 [file 12910_2024_1130_MOESM1_ESM.docx]

Semi-structured Interview Guide

This study aims to explore the anticipated and unanticipated ethical implications of introducing ICU virtual visits during the pandemic.

Estimated duration: 30-40 minutes

Probing question examples:

- Tell me more
- Please can you further explain that
- Please describe that in more detail

Background:

When did you start working on the life lines project?

What were you doing prior to joining the family communications team?

1. Please describe the process of carrying out a video-call in ICU
2. Were there any ethical issues you anticipated before setting up video calls in ICU?
3. Were there any ethical issues that arose from setting up video calls in ICU?
4. Can you tell me about a time you carried out a video call that went well?
5. Can you tell me about the most memorable video call you carried out?
6. What were the perceptions staff had about video calls in the ICU?
7. How did the video calls affect:

- Patient well being
- Family member well being
- Staff well being
